# Concomitant ligamentous and meniscal knee injuries in femoral shaft fracture

**DOI:** 10.1007/s10195-013-0255-x

**Published:** 2013-07-24

**Authors:** Mohammad Kazem Emami Meybodi, Morteza Jannesari Ladani, Tohid Emami Meybodi, Alireza Rahimnia, Ahmad Dorostegan, Jalil Abrisham, Habib Yarbeygi

**Affiliations:** 1 Orthopedic Surgery Department, Baghiatallah University of Medical Sciences, Tehran, Iran; 2 Shahid Sadoughi University of Medical Sciences, Jomhuri Boulevard, Yazd, Iran; 3 Orthopedic Surgery Department, Shahid Sadoughi University of Medical Sciences, Yazd, Iran; 4 Department of Physiology and Biophysics, Baghiatallah University of Medical Sciences, Tehran, Iran

**Keywords:** Femur fracture, Knee injury, Arthroscopy, Concomitant knee injury

## Abstract

**Background:**

Concomitant knee injury is a common finding in femoral fractures but can be easily missed during early management of the initial trauma. Degrees of damage to the articular structures vary considerably; from only a mild effusion to complete ligamentous and meniscal tears. Since previous reports were mostly from developed societies, this study was designed to look into characteristics of associated knee injury in a sample from Iran, to represent a developing country perspective.

**Materials and methods:**

Consecutive patients admitted to an orthopedic ward of Baqiyatallah hospital (Tehran, Iran) with diagnosis of femoral fracture were enrolled in this study between October 2008 and September 2009. In patients who met the inclusion criteria of the study, arthroscopic or open surgical examination of the knee, ADT, Lachman test, varus and valgus stress tests under anesthesia were carried out to determine the incidence of knee injury.

**Results:**

Forty patients with ipsilateral and two patients with bilateral femoral fractures were studied. Arthroscopy revealed medial meniscus injury in 12 (27 %) knees. Three (7 %) lateral meniscus injuries, 18 (40.9 %) ACL injuries and 2 (4.5 %) PCL injuries were also found. In varus and valgus stress tests, 15 (34 %) MCL and 4 (9 %) LCL laxities were noticed. The Lachman test was positive in 3 (6 %), and ADT was positive in 2 (4.5 %) patients.

**Conclusions:**

Based on our observations, concomitant ligamentous and meniscal knee injury is a common finding in femoral shaft fractures and rates of these injuries are generally in concert with reports from developed nations.

## Introduction

It is estimated that the annual incidence of femoral shaft fracture is 9.9 fractures per 100,000 person-years [1]. The records of fractures in England and Wales during the period 1988–1998 revealed that femur/hip fracture is the second most common fracture in women (17.0 per 10,000 person years) and seventh most common in men (5.3 per 10,000 person-years) [2]. Femoral fractures have two critical peaks of distribution: (1) young adults (from 15 to 34 years of age), and (2) elderly (over 70 years of age) [3]. High energy trauma is the main cause of fractures in younger populations, whereas low energy trauma accounts for most of the cases in people aged 60 or older [4]. An abundance of studies have reported that the incidence of femoral fracture increases with age [5–8].

Simultaneous knee injury is frequently seen in patients with femoral fractures [9]. These accompanying injuries can be easily missed during early management; since the physician or orthopedic’s attention is usually focused on the initial injury [10]. In the past 40 years, different studies have been published and focused on knee injury concomitant with femoral fracture. Most have described ligamentous damage [9, 11–16] and in the past few years some have also studied concomitant meniscal injuries [10, 17, 18].

While a relatively rich body of evidence exists regarding types and characteristics of concomitant knee injuries with femoral injuries, almost all of these reports are confined to the developed world and observations from the perspective of a developing country are lacking. Therefore, in this study, we aimed to investigate for the first time, the prevalence, types, and features of damaged knee accompanying femoral fractures. Additionally, statistical analyses were conducted to look into the correlations between fracture characteristics and knee injury.

## Materials and methods

### Patients

A cross-sectional study of patients admitted to an orthopedic ward of Baghiatallah Hospital (Tehran, Iran) with diagnosis of femoral fracture between October 2008 and September 2009 was initiated. The inclusion criteria were as follows: (1) open or closed femoral fracture (those with simultaneous tibial fracture were also included); (2) no history of previous injury to the knee. All patients were given written informed consent and the local ethics committee at Baghiatallah Hospital confirmed the study protocol. The present study was designed in accordance with the latest Declaration of Helsinki for investigation on human subjects.

### Clinical assessment

After femoral fixation with intramedullary rod placement, external fixation, or plate fixation, all patients underwent a thorough physical examination of the involved limb including varus and valgus stress tests, Lachman test, and anterior drawer test (ADT), under anesthesia. Clinical assessments were done to compare with the contralateral knee. In cases where the knee had to be exposed for retrograde nailing, a direct examination of the involved joint was also performed. Therefore, in this group of patients there was no need to perform arthroscopic evaluation. Instead, direct examination was done.

The anterior drawer test was performed with the patient lying supine. Hips were flexed, knees were flexed to 90°, with the feet placed flat on the table. The tibia was pulled forward on the femur by placing hands around the tibia. When the tibia moved forward more than 6 mm on the femur, the test considered as positive [19]. The Lachman test was done with the patient lying supine; the patient’s knee at 15° of flexion and an external rotation was performed, stabilizing the femur with one hand as the tibia moved forward. Presence of a mushy or soft endpoint when the tibia was moved forward on the femur was considered as positive [19].

Valgus and varus stress tests were performed while the patient was lying supine and the knee was in complete extension. The examiner placed one palm against the lateral aspect of the patient’s knee at the joint line.

Additionally, all knees were examined using arthroscopy. Arthroscopy was carried out with patients lying down in the supine position and anterolateral and anteromedial portals of entry were used. All clinical assessments were carried out by a single trained orthopedic.

### Statistical analysis

Statistical analyses were done using SPSS software package version 17.0 for Windows (SPSS Inc. Chicago, IL, USA). Association between categorical variables was investigated using a Chi square test for contingency tables. A Fisher exact test was used where appropriate. In all instances, a *p* value of less than 0.05 was considered statistically significant.

## Results

A total of 47 patients with femoral fractures were admitted to an orthopedic ward between October 2008 and September 2009; however, since three had previous ACL tears and two did not undergo arthroscopic examination, only 42 patients met the study criteria. Forty patients had ipsilateral femoral fracture and two had bilateral involvement. A total of 44 knees were enrolled in this study. Mean age of the study participants was 29.2 years (ranging from 17 to 48) with men constituting 86.4 % (*n* = 38) of the study population. The right and left femur were fractured in 24 (54.5 %) and 20 (45.5 %) cases, respectively. The most common cause of injury was high energy trauma due to motor vehicle accident (*n* = 39). Management of femoral fracture was done using intramedullary nailing in 40 (91 %) cases, external fixation in 3 (7 %) and plate fixation in 1 (2 %) patient. From 40 patients who underwent intramedullary nailing, based on the site and type of fracture, in 16 cases the knee was opened for distal femoral nailing. These patients did not undergo arthroscopic evaluation, since the procedure made direct examination of the knee possible. The rest of patients underwent arthroscopic assessments after fixation of fractures. Characteristics of femur fractures are presented in Table 1.

**Table 1:** Characteristics of femur fractures in the study population

	*n* (%)
Cause of fracture	
Motor vehicle accident	39 (89 %)
Falling	4 (9 %)
Gunshot	1 (2 %)
Laterality	
Right side only	24 (54 %)
Left side only	20 (46 %)
Classification of fractures^a^	
Type I	11 (25 %)
Type II	13 (29 %)
Type III	12 (28 %)
Type IV	8 (18 %)
Localization of injury	
Proximal third	8 (18 %)
Middle third	16 (36 %)
Distal third	20 (46 %)
Management	
Intramedullary rod placement	40 (91 %)
External fixation	3 (7 %)
Plate fixation	1 (2 %)

^a^According to Winquist and Hansen classification of femoral shaft fracture [25]

Using arthroscopy or direct examination, a medial meniscal tear was detected in 12 (27 %) knees. In seven cases the tear was in the central third of the meniscus and in the remainder, the posterior third was involved. Lateral meniscal tear was found in three (7 %) knees. Eighteen (40.9 %) knees had ACL injury, of which only two were complete and the other 16 were incomplete injuries. Two (4.5 %) knees had incomplete PCL tears. Chondral injury was found in 20 (45.4 %) knees, 12 (27 %) were medial, six (13 %) were lateral, and the other two (4.5 %) had injuries at the posterior surface of the patella.

Varus and valgus stress tests revealed that 15 (34 %) and four (9 %) knees had MCL and LCL laxity, respectively. The Lachman test was positive in three (6 %) knees. ADT was positive in two (4.5 %) knees. In total, 21 (47.6 %) knees had injury; with 14 (31 %) knees presenting with significant effusion. Incidences of knee ligamentous and meniscal injuries are presented in Table 2.

**Table 2:** Incidence and types of knee injuries

	*n* (%)
Arthroscopic findings	
Medial meniscal tear	12 (27 %)
Central third tear	7 (15 %)
Posterior third tear	5 (11 %)
Lateral meniscal tear	3 (7 %)
ACL injury	18 (40.9 %)
Complete injury	2 (4.5 %)
Incomplete injury	16 (36 %)
PCL injury	2 (4.5 %)
Complete injury	0
Incomplete injury	2 (4.5 %)
Chondral injury	20 (45.4 %)
Medial	12 (27 %)
Lateral	6 (13 %)
Posterior surface of patella	2 (4.5 %)
Stress tests	
MCL laxity	15 (34 %)
LCL laxity	4 (9 %)
Positive Lachman	3 (6 %)
Positive ADT	2 (4.5 %)

Men were significantly more likely to suffer a high energy trauma (*p* = 0.015). No association was found between site or type of fracture and gender (*p* = 0.35 and *p* = 0.56, respectively). Also, there was no significant correlation between site of fracture and ACL injury (*p* = 0.2), PCL injury (*p* = 0.3), or meniscal involvement (*p* = 0.7). Type of femoral fracture was associated with ACL and medial meniscal injury (*p* = 0.031 and *p* = 0.046, respectively). On the other hand, neither PCL tear nor lateral meniscal trauma were linked to fracture type (*p* = 0.439 and *p* = 0.736, respectively).

Distribution of chondral injuries were significantly different in various types of trauma (*p* = 0.02). However, no association was found between chondral injuries and ACL, PCL, LCL and MCL injury (*p* = 0.7, 0.38, 0.51).

In one patient who had concomitant femoral and tibial fracture (floating knee), proximal intramedullary nailing was done. In this case, the cause of fracture was high energy trauma and resulted in ACL and medial meniscus injury along with chondral lesions.

Although different types of ligamentous and meniscal injury were observed in our patients, arthroscopic or direct evaluation did not disclose any vascular or nerve injury.

## Discussion

High energy trauma can be the cause of femoral shaft fractures and also simultaneous pathology in the ipsilateral knee. As expected, a high percentage of patients examined in this study had significant knee injury. The most common site of injury was ACL. Chondral injury was also a common finding.

Pederson and Serra [14] were the first to report that serious injury to the major ligaments of the knee may occur in association with fractures of the femur. They examined six patients with fractures of the midshaft of the left femur, and fractures of the shaft of the left tibia and fibula. The main cause of the injury in their patients was car accident. Five of the six patients suffered from rupture of the right collateral ligament [14].

Walker and Kennedy [13] reviewed 52 patients with 54 midshaft femoral fractures. A high incidence (48 %) of ipsilateral knee ligament damage was reported. In their study motor vehicle, athletic injuries, and falls accounted for all of the cases.

Walling et al. [15] evaluated 24 American patients with fractures of the femoral shaft. In this observation, 33 % had injuries of the ligaments of the ipsilateral knee.

Moore et al. [9] investigated 309 patients with 320 diaphyseal femur fractures. Contrary to most of the literature, only 17 (5.3 %) patients with unilateral shaft fractures of the femur had ipsilateral knee ligament damage. Also, they reported that there was no relationship between specific ligament damage and the cause of the injury or level of fracture.

Szalay et al. [16] inspected 110 Australian patients with 114 femoral shaft fractures. In 27 % of patients ligament laxity was detected. They also inspected another 33 patients with 34 ipsilateral femoral and tibial fractures. In the latter group the results were more pronounced: 53 % of patients had knee ligament laxity; leading to the conclusion that knee ligament injury is more common in simultaneous femoral and tibial fractures than in single femoral fractures.

Vangsness et al. [17] examined 47 patients with femoral fractures using arthroscopy, focusing on meniscal injuries. The common cause of injury was blunt trauma. Examinations were done after intramedullary fixation. In this report, 12 and 13 patients suffered from medial and lateral meniscal injuries, respectively. Bilateral injuries were found in two patients. Moreover, ligamentous laxity was detected in 49 % of patients. The incidence of meniscal injury was no different in patients with or without ligamentous injury. The incidence of meniscal injury in this study (57 %) was relatively higher than our result (34 %).

De campos et al. [18] examined forty adults with femoral shaft fractures and no history of previous knee injury. The incidence of ligamentous laxity was 52.5 %. The most significant arthroscopic findings were anterior cruciate ligament injury that was presented in 21 patients. Three patients had posterior cruciate ligament injury. Five medial and eight lateral meniscus tears were also noted. Consistent with our findings, ACL injury was more common than PCL injury.

In a recent investigation, Blacksin et al. [20] assessed 34 femoral fractures with magnetic resonance imaging. Imaging was done, on average, 2.5 days after injury. Assessment revealed meniscal tears in 27 %, medial collateral ligament injury in 38 %, and posterior cruciate ligament injury in 21 % of the patients. After imaging was performed, they compared MRI results with clinical examinations. The Lachman test was positive in two patients, but MRI showed no evidence of anterior cruciate ligament injury in these patients. Comparing physical examinations and MRI findings shows that there is no correlation between these two methods.

Auffrath et al. [10] reviewed 103 Austrian patients with femoral shaft fractures during 2000–2007. They excluded patients who had obvious knee injury at the time of admission; their goal was to investigate the number and severity of knee injuries that remain undetected at the time of admission. Fifty-three patients with 55 midshaft femoral fractures were included, based on their criteria. They found three injuries: one was partial tear of the posterior cruciate ligament, and two were medial meniscus injuries.

Many of the correlations assessed did not reach statistical significance. This is in part due to the relatively small sample size in our study. Future studies with large enough sample sizes are paramount to elucidate possible risk factors for articular damage accompanying thigh bone fractures. In our study, the Lachman test was negative in the majority of subjects who suffered ligamentous injuries. This is likely due to the Lachman test being unable to detect partial tears. Sixteen out of 18 injuries observed herein were of a partial nature, and the Lachman test was only positive in one. It has been previously stated by different authors that it is difficult to identify partial ACL tears in a physical examination; additional assessment using MRI or arthroscopy is needed for detection of these injuries [21, 22]. This discrepancy further highlights the need for careful evaluation of affected knees in patients with femoral fractures, even in the face of an evidently normal physical examination, since the Lachman test has a limited ability in detecting partial tears.

Traffic and road accidents in Iran are a public health concern and their prevention remains a health priority. Accidents in Iran are the second most common cause of mortality in the country, trailing only behind cardiovascular diseases [23]. Individuals involved in road accidents are often of young age and tend to suffer significant musculoskeletal injuries. Based on available reports, the most prevalent bone injuries due to accidents are tibial and femoral fractures, accounting for 49.8 and 19.9 % of musculoskeletal injuries, respectively [24]. Here, we have shown that these fractures often are accompanied by ligamentous injuries that without a thorough and careful assessment of the injured organ would go undiagnosed. Given the immense burden that lower limb fractures impose on the individual, and lifetime disability and productivity loss associated with it, proper early management of the fracture is of utmost priority.

In summary, for the first time we investigated the presence and features of simultaneous knee injuries in femur fractures in a well-defined sample of Iranian adult patients. Our observations confirm the fact that knee injuries are a rather common finding in femoral fracture cases; therefore, careful examination of the affected joint with aid of other imaging modalities or arthroscopic examinations can result in early diagnosis, management and repair of the injured soft tissue.
